# A Case of a Huge Granulomatous Epulis in the Mandibular Alveolar Region

**DOI:** 10.1155/crid/5558231

**Published:** 2026-01-08

**Authors:** Yu Hara, Kunio Yoshizawa, Junya Furukawa, Atsuya Ishiyama, Riku Kohara, Karen Gomi, Akinori Moroi, Koichiro Ueki

**Affiliations:** ^1^ Department of Oral and Maxillofacial Surgery, Division of Clinical Medicine, Interdisciplinary Graduate School, University of Yamanashi, Chuo, Yamanashi, Japan, yamanashi.ac.jp

**Keywords:** differential diagnosis, granulomatous epulis, histopathological diagnosis, mandibular tumor

## Abstract

Epulis is a general diagnostic term for benign localized tumors that occur in the gingiva. Clinical diagnosis is usually straightforward based on the location and morphology of the lesion; however, large lesions may mimic malignancy. We report a case of a large granulomatous epulis in the mandibular alveolar region. A male in his 60s presented with swelling in the right mandible. Examination revealed a well‐defined, pedunculated soft tissue mass in the right mandibular gingival region. Then, 1 month after the initial visit, the lesion (55 × 35 × 20 mm) was resected under general anesthesia. Histopathological examination confirmed granulomatous epulis, and the patient has remained free of recurrence after 6 months of follow‐up.

## 1. Introduction

Epulis is a general term describing benign localized growths of the gingiva, often resulting from chronic irritation such as sharp tooth edges, poor prostheses, or neglected dental treatment. Histopathologically, granulomatous epulis consists of proliferative granulation tissue covered by mucosal epithelium with inflammatory cell infiltration and edema.

We report a case of a large granulomatous epulis arising from the mandibular alveolar region. Although not rare, this case was initially suspected to be malignant due to its size and surface irregularity. The diagnostic process and final histopathological confirmation are discussed.

## 2. Case Presentation

A male in his 60s presented in October 2024 with swelling of the right mandibular gingiva. His medical history included hypertension and diabetes mellitus, with no relevant family or medication history. The patient had first noticed the swelling approximately 8 months earlier but did not seek treatment because it was painless.

**Figure 1:**
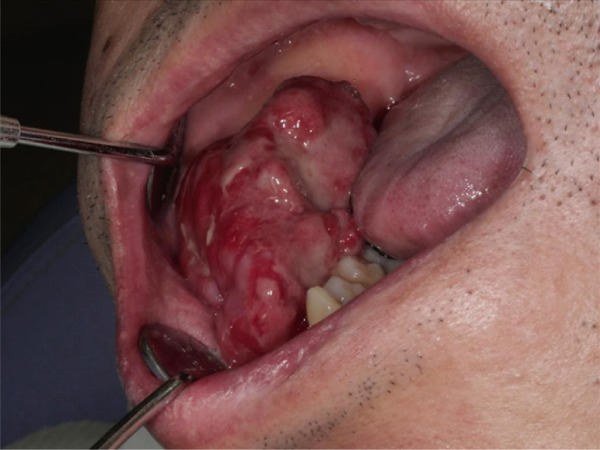
Intraoral photograph at the initial examination showing gingival swelling of the right mandible with an irregular surface.

Extraoral examination revealed preserved facial symmetry. Intraorally, a pedunculated, reddish mass with an irregular surface was noted in the right mandibular gingiva, raising suspicion of malignancy (Figure 1).

**Figure 2:**
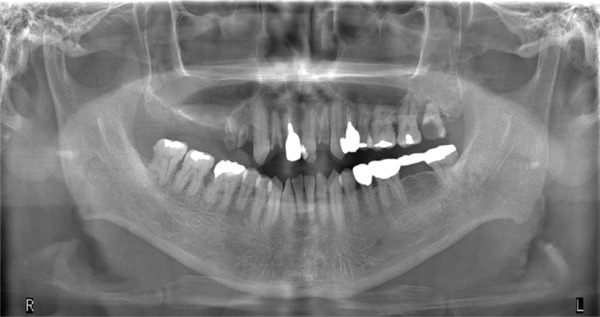
Panoramic radiograph showing no bone resorption.

**Figure 3:**
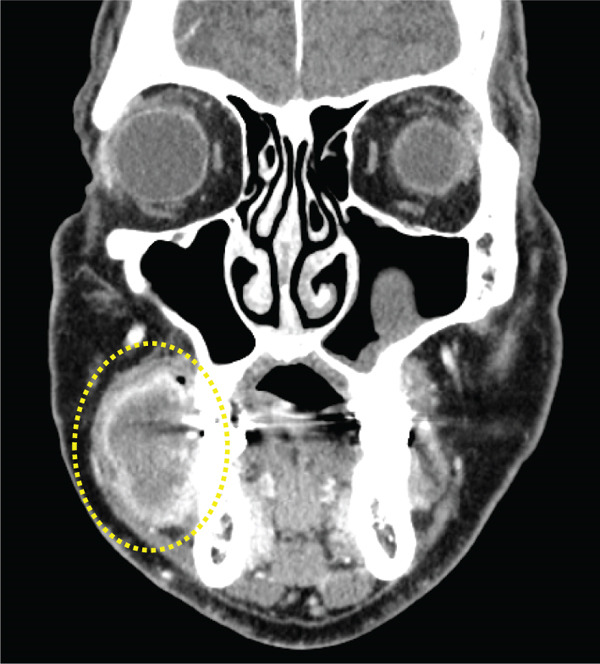
Contrast‐enhanced CT image showing no calcification or bone invasion.

Panoramic radiography showed no evidence of bone resorption (Figure 2). Contrast‐enhanced CT imaging revealed a soft tissue mass without calcification or bony invasion (Figure 3). Based on these findings, a provisional diagnosis of a right mandibular tumor was made.

**Figure 4:**
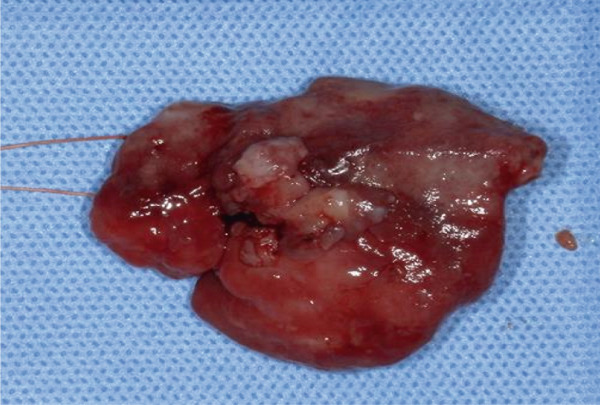
Excised specimen measuring 55 × 35 × 20 mm with a smooth surface.

**Figure 5:**
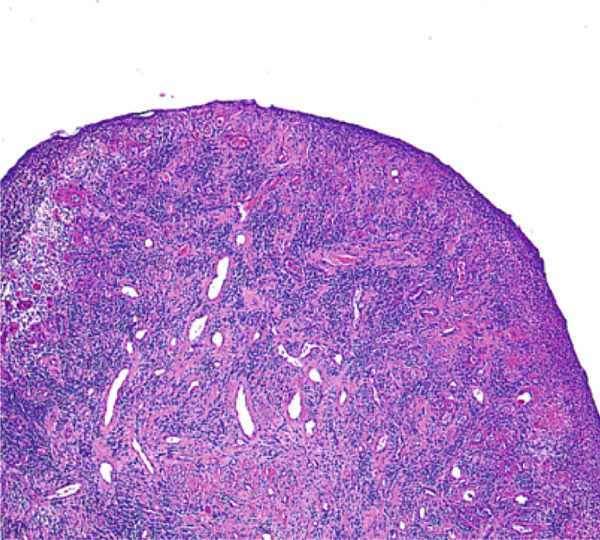
Histopathological image (H&E stain, × 40) showing squamous epithelium with inflammatory infiltration and granulation tissue formation.

In November 2024, the lesion was completely excised under general anesthesia using a scalpel and electrocautery (Figure 4). The surgical wound was sutured, and healing was uneventful. Histopathological analysis revealed squamous epithelial tissue with inflammatory cell infiltrates, including lymphocytes, plasma cells, and neutrophils, with fibrosis and granulation tissue formation in the stroma (Figure 5).

A final diagnosis of *granulomatous epulis* was made. At 6 months postoperatively, the patient showed good healing with no evidence of recurrence.

## 3. Discussion

Epulis is not a true neoplasm but a reactive benign lesion. Among its variants, granulomatous epulis frequently presents with ulcers, erythema, or an irregular surface that can mimic malignant tumors, making it commonly encountered. [1] In this case, the lesion′s large size (55 × 35 × 20 mm) and irregular surface initially raised clinical suspicion of malignancy. While epulis is relatively easy to diagnose clinically based on location and characteristics, large lesions or those with unusual surface features can sometimes clinically mimic malignancy at first glance. Indeed, cases have been reported where lesions suggestive of epulis were histopathologically found to be metastatic carcinoma to the gingiva [2], malignant salivary gland tumors [3], squamous cell carcinoma, or Burkitt lymphoma [4]. Therefore, when a neoplastic lesion cannot be ruled out, performing a preoperative biopsy is crucial. Findings suggestive of a tumor include the following: (1) large lesion size, (2) rapid growth, (3) broad base, (4) alveolar bone resorption, (5) multinodular appearance, (6) granular texture, (7) ulceration, and (8) easy bleeding. In our case, the large lesion size meant a neoplastic lesion could not be excluded. However, imaging studies showed no evidence of infiltration or destruction. A preoperative biopsy was performed, and histopathology confirmed a benign inflammatory lesion, leading to excision. Performing a preoperative biopsy is crucial [5].

While this case is not particularly rare, the reasoning process in the diagnostic pathway holds educational value. The absence of pain and the patient′s poor oral hygiene likely contributed to the delayed diagnosis and enlargement of the lesion. Therefore, regular follow‐up and oral hygiene management are necessary. Clinicians encountering large gingival masses should consider a broad differential diagnosis including pyogenic granuloma, marginal osteoclastoma, marginal giant cell granuloma, and squamous cell carcinoma.

Accurate diagnosis requires correlating clinical findings, imaging findings, and histopathological findings, followed by removal of the causative stimulus. Reporting detailed diagnostic reasoning enhances the educational value of such cases and helps clinicians distinguish benign reactive lesions from malignant tumors.

## 4. Conclusion

Large granulomatous epulis lesions may mimic malignancy due to their size and irregular appearance. This case emphasizes the importance of systematic diagnostic assessment combining clinical, imaging, and histopathological findings to ensure correct diagnosis and management.

## Ethics Statement

This case report adheres to the Declaration of Helsinki, and written informed consent was obtained from the patient.

## Conflicts of Interest

The authors declare no conflicts of interest.

## Funding

No funding was received for this manuscript.

## Data Availability

Data sharing is not applicable to this article as no datasets were generated or analyzed during the current study.
